# Physical Health and Mental Health Comorbidities of People With Functional Neurological Disorders Referred to a Community Neuropsychiatry Service Pre-& Post March 2020 Lockdown Due to COVID-19

**DOI:** 10.1192/bjo.2022.421

**Published:** 2022-06-20

**Authors:** Verity Williams, Oluwaseun Olaluwoye, Alan Dunlop, Rafey Faruqui

**Affiliations:** 1 Kent and Medway NHS and Social Care Partnership Trust, Ashford, United Kingdom; 2 Kent and Medway Medical School, Canterbury, United Kingdom; 3 Centre for Health Services Studies, University of Kent, Canterbury, United Kingdom

## Abstract

**Aims:**

Health comorbidities contribute significantly to the development and maintenance of illness in patients with Functional Neurological Disorder (FND). As part of a service evaluation project, we investigated the physical and mental health comorbidities of people referred to a community neuropsychiatry service in East Kent, in one-year periods preceding and following the March 2020 lockdown due to COVID-19.

**Methods:**

We included all people accepted to the service between 23rd March 2019 and 23rd March 2021, where the reason for referral was Functional Neurological Disorder (FND) or Non-Epileptic Attack Disorder (NEAD). Referrals to the service for other reasons were excluded, as were declined referrals. Routinely collected data sources were reviewed and data stored in anonymized fashion. Data were analysed using Statistical Package for Social Sciences (SPSS).

**Results:**

Total number of referrals for FND in the 2-year period was 260, with 161 referrals for NEAD and 99 for other FND.

In the pre-lockdown period, 163 patients were referred due to FND (101 with NEAD, 62 for other FND). There were fewer FND referrals in the post-lockdown period: 60 referrals for NEAD and 37 for other FND. The majority were female (74% pre-lockdown, 81% post-lockdown). Where ethnicity was recorded, White British was the most common (94% pre-lockdown, 90% post-lockdown), with a small number of people from other ethnic groups (3.5% White Other, 1.4% BAME, 1.4% Mixed pre-lockdown; 5.4% White Other, 3.2% BAME and 1.1% Mixed post-lockdown). Ethnicity was not specified in 21 cases (13%).

Of the pre-lockdown group, 15 patients had prior contact with Child and Adolescent Mental Health Services (9%), with 7 patients (7%) in the post-lockdown group. Many patients had previous contact with mental health services (47% pre-lockdown, 53% post-lockdown). The majority of patients had at least one physical illness (69% pre-lockdown, 73% post-lockdown). Most had 1–3 physical comorbidities but 9% (pre-lockdown, 7% post-lockdown) had more than 4. Fibromyalgia (14% pre-lockdown, 12% post-lockdown), chronic pain (23% pre-lockdown, 21% post-lockdown), and epilepsy (11%, 9%) were common. Over 90% had psychiatric illness in both periods. Most patients had 1–3 psychiatric illnesses; a few had more than 4 (6.1% pre-lockdown, 1.4% post-lockdown). Depressive disorder was the most common comorbidity in both groups (41% pre-lockdown, 44% post-lockdown), followed by anxiety (35% pre-lockdown, 36% post-lockdown). PTSD was present in 8% pre-lockdown and 8.2% post-lockdown.

**Conclusion:**

Physical and psychiatric comorbidities are common in people with FND; multidisciplinary working and liaison between services is crucial for care of these patients.

